# Validation of a German version of the International Hip Outcome Tool 12 (iHOT12) according to the COSMIN checklist

**DOI:** 10.1186/s12955-016-0407-9

**Published:** 2016-01-08

**Authors:** Florian Baumann, Daniel Popp, Karolina Müller, Michael Müller, Paul Schmitz, Michael Nerlich, Stefan Fickert

**Affiliations:** Department of Trauma Surgery, Regensburg University Medical Center, Regensburg, Germany; Center for Clinical Studies, Regensburg University Medical Center, Regensburg, Germany; Medical Faculty Mannheim, University Medical Centre Mannheim, Heidelberg University, Heidelberg, Germany; Sporthopaedicum Straubing, Straubing, Germany

**Keywords:** Patient reported outcome (PRO), Hip arthroscopy, Femoro-acetabular impingement syndrome (FAI), Responsiveness, German International Hip Outcome Tool 12 (iHOT12), COSMIN checklist

## Abstract

**Background:**

Patient Reported Outcome (PRO) measurements have become an important tool to evaluate disease-related quality of life. The “International Hip Outcome Tool” (iHOT12) is a self-administered patient-reported outcome tool, which includes questions on the patient’s symptoms, functional and sports limitations as well as social, emotional, and occupational limitations. The purpose of this study was to adapt and validate a German version of the iHOT12 according to the COSMIN checklist.

**Methods:**

In order to validate the German translation of the iHOT12, we conducted a prospective multicenter cohort study on patients with hip disorders and a score ≥4 on the modified Tegner Activity Scale (mTAS). The patients completed the German iHOT12 questionnaire and other functional scores (Hip Outcome Score, modified Tegner Activity Scale, EuroQol-5D) twice at intervals of at least two weeks. Evaluation of psychometric properties was conducted following the COSMIN checklist for validation of health status measurement instruments. The methodical testing for reliability included internal consistency, test-retest reliability, and measurement error. For testing of validity, we analyzed construct validity, hypotheses testing, interpretability and responsiveness.

**Results:**

Between December 2013 and December 2014, eighty-three consecutive patients completed both questionnaires and were available for data analysis. Cronbach’s alpha was 0.94 (95 %-CI: 0.91, 0.95) confirming internal consistency and test-retest reliability of the iHOT-12 was high with an ICC = 0.94 (95 %-CI: 0.89, 0.97). All a priori hypotheses were confirmed. Further, no relevant floor- or ceiling effects occurred. The iHOT12 showed good responsiveness with a minimal important change (MIC) under 14 points.

**Conclusions:**

The German translation of the iHOT-12 is a reliable, valid, and responsive tool for the evaluation of disease-related quality of life in active patients with a hip disorder. We could show that the minimal important change, a change of health condition the patient discerns, is less than 14 points in the iHOT12 scale.

## Background

Patient-reported outcome (PRO) measurements have become an important tool to evaluate activities, limitations in everyday life and the quality of life. Conventional questionnaires focus on patients with osteoarthritis or undergoing hip arthroplasty with a limited activity level [1–4]. Over the last decade, the better understanding of specific hip pathologies has evolved joint preserving procedures such as hip arthroscopy or surgical hip dislocation [5]. The mainly young and active patients undergoing these joint preserving procedures have different expectations and aims of their surgery. Conventional outcome tools do not reflect their situation adequately [6–8]. Further therapeutic innovations are likely to achieve only minor improvements. With a high discriminatory power, these instruments can reveal minor outcome differences.

Recently, the Multicenter Arthroscopy of the Hip Outcomes Research Network (MAHORN) study group developed a new PRO questionnaire with special concern for young, active patients with hip disorders [9]. Compared to other questionnaires, the iHOT includes inquiries of limitations in social interactions, emotional issues and working life. The original score consisted of 33 items and is validated in English, Spanish, and German language [9–11]. There is also a short version of 12 items available in English and Spanish language [12]. The iHOT has shown a high reliability and validity.

Studies evaluating measurement properties have to meet a high methodological quality. The COSMIN checklist (COnsensus based Standards for the selection of health status Measurement INstruments) is a consensus-based checklist to evaluate the methodological quality of studies on measurement properties of health status measurement instruments based on an international Delphi study in 2010 [13].

The purpose of this study was to validate a German version of the iHOT-12 according to the COSMIN checklist and compare the psychometric properties of the short version (iHOT12) to the extended version (iHOT33).

## Methods

### International Hip Outcome Tool (iHOT)

In 2012, the Multicenter Arthroscopy of the Hip Outcomes Research Network (MAHORN) developed the International Hip Outcome Tool, a self-administrated questionnaire originally comprising of 33 items. [9]. The patient is asked to consider the problems of the past month and to indicate the severity on a 100 mm horizontal line (visual analogue scale) by marking it with a slash. Each question has equal weight so that the mean of all questions amounts to the score result ranging from 0 to 100. A score of 100 indicates full function and no symptoms, whereas a score of zero signifies maximum limitations and extreme symptoms. There is also a short form of 12 items available (iHOT-12). The iHOT has shown a high internal consistency, construct validity, and responsiveness [8, 9, 14–16].

### Adaption of the iHOT-12

The translation of the iHOT-12 into German was carried out following the guidelines of the American Academy of Orthopedic Surgeons (AAOS) Outcomes Committee [17]. According to these guidelines, an informed and an uninformed translator translated the iHOT-12 from English into German independently. After consolidation of both translations, a German linguist reviewed the German version of the questionnaire. Two native speaking translators (informed and uninformed) re-translated this German version into English. This version was verified for consistence. Finally, the German questionnaire was tested for comprehensibility in 20 patients with a hip disorder. The translation process was supervised and documented in a survey report.

### Validation study

We performed a prospective multicenter study to evaluate reliability, validity, and responsiveness of the German version of the iHOT-12. Inclusion criteria were a history of a hip disorder, a score of ≥4 on a modified Tegner Activity Scale [18], and sufficient reading and comprehension capacity. Patients were excluded if they had a disorder of the back or the contralateral lower extremity, a score less than 4 on the modified Tegner Activity Scale, a mental disorder, or a lack of informed consent to participation [9]. All patients are seen in an outpatient setting. The patients primarily completed the questionnaire before seeing the orthopedist. For evaluation of test–retest reliability, the patients completed a second questionnaire after a minimum of two weeks. The patients were asked to answer the questions according to their current status and return the forms by mail. We reminded all patients who did not answer within six weeks by telephone. All patients had given their written informed consent to participate in this study. The Regensburg University Ethics Committee approved the study in November 2013 (Institutional Review Board Number 13-101-0259).

### Questionnaire

In addition to the iHOT-12, the questionnaire consisted of the following scores:

#### Hip Outcome Score (HOS)

The HOS is an established 31-item PRO tool to evaluate activities, limitations in everyday life, and quality of life of patients with a hip disorder. It comprises of two subscales on activity of daily life and sports activities. The patient is asked to answer the questions considering the past week. Scores range 0–100, higher scores represent a better function and a higher level of activity [19]. The HOS has been validated and published in German in 2011 [20].

#### Modified Tegner Activity Scale (mTAS)

The TAS is a 10 level activity scale reflecting the patient’s currently highest level of sports activity or other routine activities. Initially it was designed as a complement to other functional scores of the knee joint and is the most commonly used activity scoring tool [18]. Although there is no validation study of the hip modification of the TAS, it is also well established and widely used [9, 12, 16, 21]. A score greater than 4 was an inclusion criterion for the evolution study of the iHOT-33 by Mohtadi [9]. Hence, we included the scale to our questionnaire to achieve a similar cohort.

#### EuroQol-5D (EQ 5-D)

The EQ 5-D is a global quality of life questionnaire consisting of a 5-item assessment of the health status regarding mobility, self-care, usual activities, pain/discomfort, and anxiety/depression [22]. The second part of the EQ 5-D consists of a 200 mm analogue scale concerning the patient’s assessment of the current global health status. The EQ 5-D has been adapted to German and is validated for a number of health compromising conditions [23].

#### Subjective assessment

The patient was asked to assess his current limitations concerning function (pain, ROM, etc.), sport/leisure activities, employment/housekeeping, and social interaction/quality of life. The limitations should be estimated in percent from 0 % = no limitation at all to 100 % = maximum.

The second set of questions also included an evaluation of whether the condition of their hip joint was ‘much better’, ‘somewhat better’, ‘unchanged’, ‘somewhat worse’, or ‘much worse’ compared to the primary evaluation.

### Statistical analysis

Questionnaires with any missing data or unclear marking were excluded from the analysis. Statistical analysis was performed using the software package SPSS (Version 23, SPSS Inc., Chicago, Illinois). Unless otherwise stated, descriptive data are given as mean ± standard deviation. The level of significance was defined at *p* < 0.05 for all tests.

## Methodological testing according to the COSMIN checklist

### Reliability

Reliability is the degree to which the measurement is free from measurement error [24]. To evaluate reliability, internal consistency, test-retest reliability, and measurement error are calculated.

#### Internal consistency

Internal consistency is described as the degree of interrelatedness among items [24]. Sufficient internal consistency was assumed for a Cronbach’s α greater than 0.7 [25].

#### Test–retest reliability

Test–retest reliability is the extent to which results of the same patient in the same health condition remain unchanged over time [24]. According to the recommendation of the COSMIN manual, the retest was performed after a minimum of two weeks after outpatient consultation to avoid recollection of the answers and changes in health condition. Intraclass correlation coefficients (ICC) were calculated for all patients indicating an unchanged condition of their hip joint since the primary evaluation. For an ICC greater than 0.7 sufficient test-retest reliability was assumed [25].

#### Measurement error

The measurement error is the systematic and random error of a patient’s score that is not attributed to true changes in the construct to be measured [24]. The Standard Error of Measurement (SEM) was calculated using the formula SD / √1-ICC (SD = Standard Deviation; ICC = Intraclass correlation coefficient) [25]. The smallest detectable change (SDC) reflects the smallest individual change in score that can be interpreted as a real change. It was calculated by the SEM × 1.96 × √2/√n [25].

### Validity

Validity is the degree to which a questionnaire measures the construct it purports to measure [24].

#### Construct validity

Since there is no gold standard in the measurement of PRO, validity is determined by assessing construct validity. Construct validity is the degree to which the scores of a questionnaire are consistent with questionnaires measuring the same construct. To validate the German translation of the iHOT12, Spearman’s correlation coefficient was calculated between the iHOT12 and the other functional scores as well as the subjective rating by the patient. For a correlation coefficient r < 0.3 a poor and for r > 0.7 a good correlation was assumed.

#### Hypothesis testing

To analyze construct validity we tested a priori hypotheses [24]. We hypothesized that the iHOT12 would correlate well with the other subjective scales like the HOS and the EQ-5D. Therefore, we expected a Spearman’s correlation coefficient r > 0.7. We expected a low correlation (r < 0.3) between the iHOT12 and the mTAS. We correlated the subjective global rating of change (GRC) with the mean difference between the iHOT12 scores at T2-T1. We hypothesized that the changes in the iHOT12 score would correlate with the subjective evaluation of the patient [21, 26].

#### Responsiveness

Responsiveness is the ability of a questionnaire to detect a change over time in the construct to be measured [24]. According to Terwee et al. [25], responsiveness was demonstrated by comparing the smallest detectable change (SDC) to the minimal important change (MIC). Responsiveness was confirmed if the SDC < MIC. Additionally we used an anchor-based method to evaluate responsiveness [27]. At T2 the patients were asked to rate whether the current condition of their hip joint was ‘much better’, ‘somewhat better’, ‘unchanged’, ‘somewhat worse’, or ‘much worse’ compared to the condition of the primary evaluation.

#### Interpretability

**Fig. 1:**
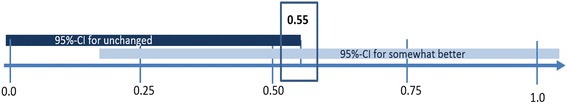
Estimation of the effect size by indication of the 95-CI for unchanged and somewhat better subgroups

Interpretability is the ability to transform a qualitative effect into a quantitative score [24]. The minimal important change (MIC) was estimated by dividing the standard deviation (SD) by two as described by Norman et al. [28]. The effect size (ES) was calculated by the mean change of the score/SD. The 95 %-CI of the effect size of the “somewhat better” group was compared to the ES of the “unchanged” group to estimate the true MIC (Fig. 1).

Another quality criterion for content validity is the absence of floor and ceiling effects. If more than 15 % of patients score highest (100) or lowest (0) value in the iHOT12, extreme outcome values might not be represented adequately [25].

## Results

### Demographic data and generalizability

Between December 2013 and December 2014 eighty-three patients completed both questionnaires and were available for data analysis. The cohort comprised of 24 women (29 %) and 59 men (71 %). The mean age was 33.7 ± 11.8 years (range 14–63). Demographic data and diagnosis-related score results are provided in Table 1. The second questionnaire was completed on average 28.5 ± 31.7 days (range 14–194) after the first. Missing items were found in 92 of 5146 items in total (1.8 %). Questionnaires containing missing items or unclear marking were excluded from the analysis. Missing items occurred randomly, there was no accumulation of missing items in any unit of the questionnaire.

**Table 1:** Demographic data and diagnosis related score results

Diagnosis	Number of patients	Mean age	Gender		iHOT-12	HOS	mTAS
			male	female			
FAI	31 (37.3 %)	28.5 (±9.6)	26	5	54.2 (±19.7)	81.6 (±12.6)	5.9
Osteoarthritis	16 (19.3 %)	47.4 (±9.0)	12	4	52.7 (±29.4)	76.9 (±17.0)	4.3
Hip dysplasia	13 (15.7 %)	24.7 (±8.2)	5	8	45.7 (±15.6)	78.9 (±10.2)	5.6
Muscular imbalance	6 (7.2 %)	31.7 (±11.1)	3	3	65.8 (±18.9)	85.2 (±6.9)	5.3
Not specified	17 (20.5 %)	37.4 (±7.7)	13	4	63.9 (±40.4)	82.2 (±23.5)	4.9
Total	83 (100 %)	33.7 (±11.8)	59	24	55.8 (±27.0)	80.9 (±15.7)	5.3

### Reliability

A Cronbach’s alpha of 0.94 (95 %-CI: 0.91, 0.95) showed excellent internal consistency for the iHOT12. The intraclass correlation coefficient (ICC) was 0.94 (95 %-CI: 0.89, 0.97) for all patients indicating an unchanged condition of their hip joint since their primary evaluation (*n* = 46). The overall SEM was 6.75. Hence, the smallest detectable change (SDC) reflecting the smallest individual change in score that can be interpreted as a real change was 2.76.

### Validity

**Fig. 2:**
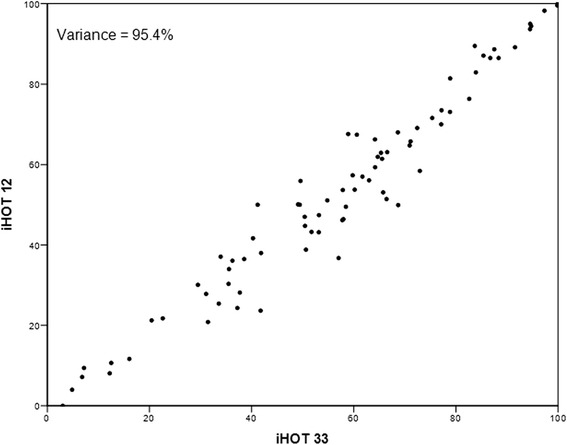
Relation between IHOT 33 and iHOT 12 scores for validation data

**Fig. 3:**
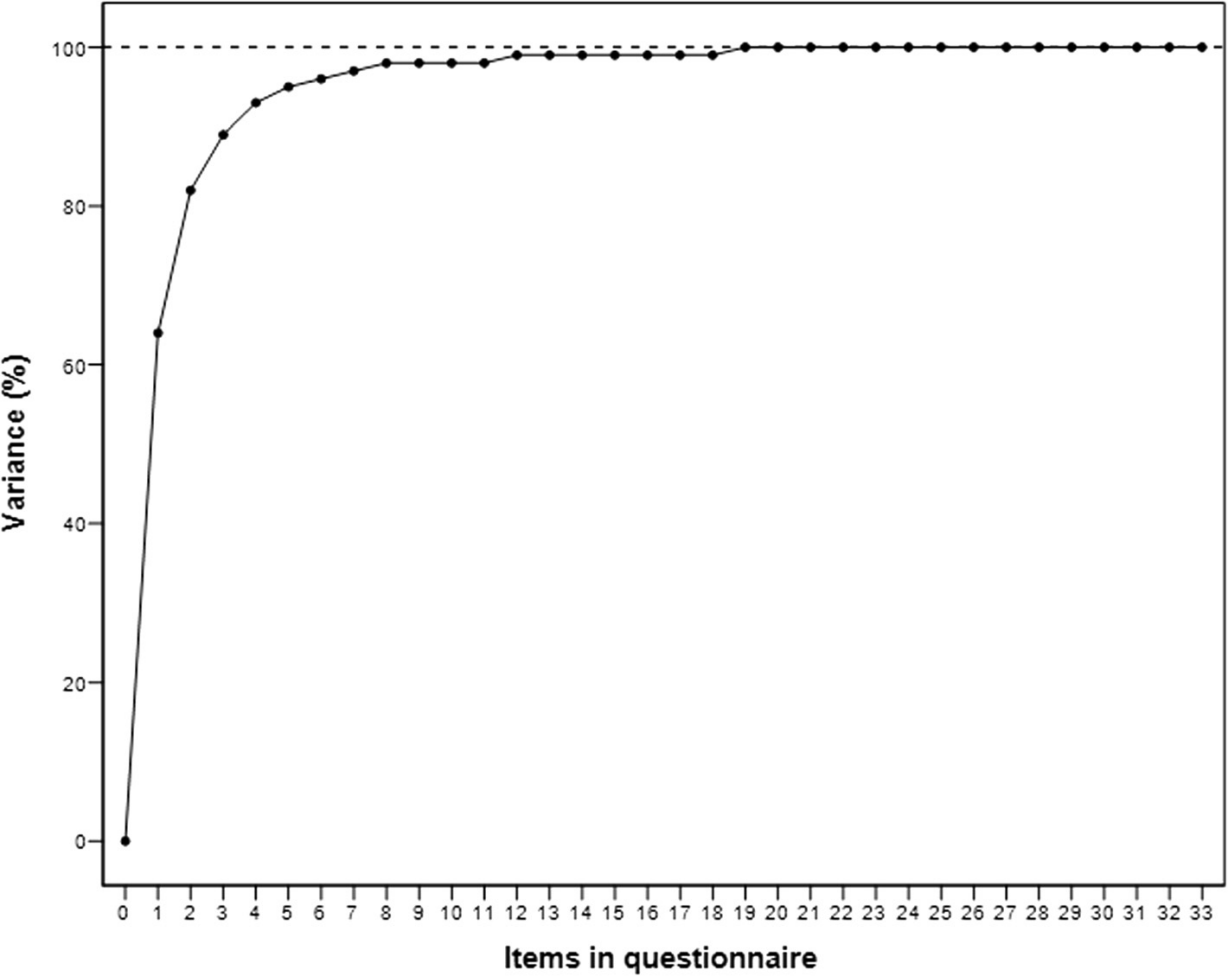
Variance (information) accounted for, expressed as a percentage, by the inclusion of increasing number of items in the questionnaire

**Fig. 4:**
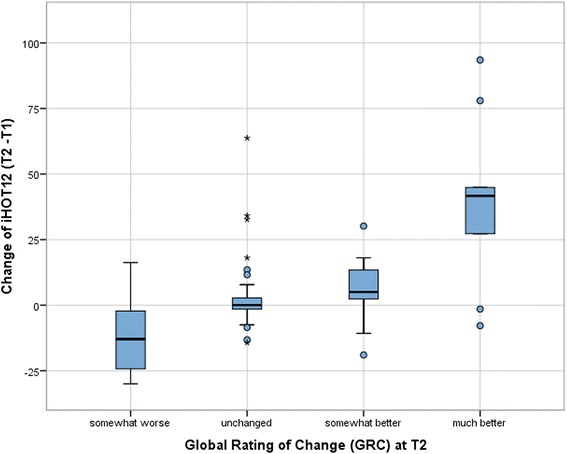
Boxplots of the iHOT12 for global rating of change categories

**Fig. 5:**
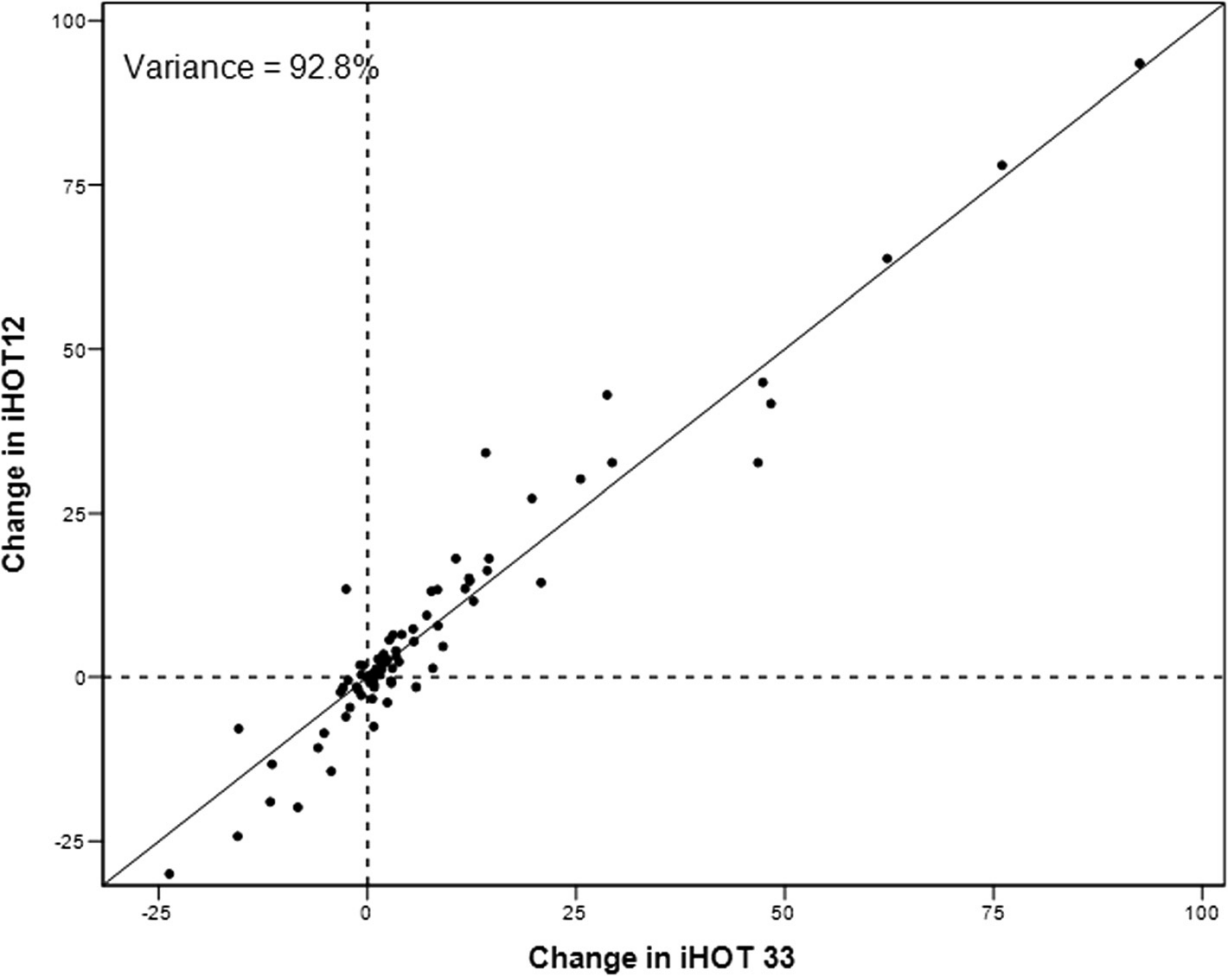
Change scores for iHOT12 and iHOT33

The assessment of the construct validity showed a good correlation between the iHOT12, HOS and EQ-5D (Table 2). There was an excellent correlation of r = 0.97 (*p* < .001) between iHOT 12 and iHOT33 (Fig. 2). Fig. 3 shows the variance accounted expressed as a percentage, by the inclusion of increasing number of items in the questionnaire. With a Spearman’s correlation coefficient of r = 0.03, there was only a poor correlation between iHOT12 and the mTAS. Therefore, all hypotheses could be confirmed. Adequate responsiveness of the iHOT12 could be demonstrated with a higher value of MIC (13.79) compared to SDC (2.76). According to the GRC dependent 95 %-CI of the ES, the estimation of ES was confirmed (Fig. 1). In addition, there was a good correlation between the global rating of change (GRC) and the mean difference between the iHOT12 scores at T2-T1 (Table 3 and Fig. 4) (r = 0.48, *p* < .001). According to the ES of 0.55 the minimal important change, a change that reflects a clinically relevant improvement is approximately fourteen points on the iHOT12. The change of scores for iHOT12 and iHOT33 is shown in Fig. 5.

**Table 2:** Spearman’s correlation coefficients between functional scores

*n* = 83	G-iHOT 12	G-iHOT 33	HOS	mTAS	EQ5D
G-iHOt 12	1	0.97 (*p* < 0.001)	0.85 (*p* < 0.001)	0.03 (*p* = 0.808)	0.78 (*p* < 0.001)
G-iHOt 33	-	1	0.87 (*p* < 0.001)	0.07 (*p* = 0.558)	0.77 (*p* < 0.001)
HOS	-	-	1	0.02 (*p* = 0.869)	0.73 (*p* < 0.001)
mTAS	-	-	-	1	0.02 (*p* = 0.835)

**Table 3:** Change sensitivity of the iHOT12 based on global rating of change

Global rating of change (GRC)	N	Mean difference	SD	Mean difference 95 %-CI		Minimum	Maximum	Effect size (ES)	ES 95 %-CI	
Somewhat worse	6	−11.01	17.08	−28.94	6.91	−30.00	16.25	-.64	−1.51	0.27
Unchanged	46	3.30	12.69	-.46	7.07	−14.33	63.75	.26	−0.03	0.55
Somewhat better	22	6.54	10.19	2.02	11.06	−19.00	30.17	.64	0.18	1.10
Much better	9	39.07	32.71	13.93	64.22	−7.83	93.50	1.19	0.30	2.04

There was no relevant floor effect for the iHOT12, since there was only one patient with a score value of zero (1.2 %). Six patients (7.2 %) scored a maximum score of 100, which is why relevant ceiling effects could be declined.

## Discussion

The present study evaluated the German version of the iHOT12. The short form of the iHOT is a quickly administrable and easy to use PRO instrument. This German version of the iHOT12 provides sufficient validity, reliability, and responsiveness for the evaluation of physically active patients with non-arthritic hip problems. There is excellent correlation of the iHOT12 to the extended version of the iHOT33.

Since femoro-actetabular impingement syndrome (FAI) has been identified as a risk factor for osteoarthritis of the hip joint, the development of joint preserving procedures has been advanced in the last decade. Due to technical improvements, hip arthroscopy has become a successful procedure to relieve pain and to restore clinical function in FAI [5, 8, 29–32]. Patient-reported outcome tools are becoming more and more important to reflect the patient’s view of the postoperative outcome and limitations in everyday life. Recently, some questionnaires were developed to evaluate the postoperative outcome in this cohort of young, physically active patients. These questionnaires mainly focus on symptoms and function in everyday life [2–4]. Most of them, however, do not give a comprehensive picture of the patients’ views. Some authors have already pointed out a discrepancy between functional results and patient satisfaction in patients undergoing hip arthroscopy [5–7, 33, 34]. Social, emotional, and occupational factors might also play an important role in the patients’ assessment of the therapy. Therefore, the Multicenter Arthroscopy of the Hip Outcomes Research Network (MAHORN) has developed an outcome measurement instrument including questions on social, emotional, and occupational limitations. The iHOT has also been cross-culturally adapted into Spanish, Portuguese and Swedish [11, 35, 36]. Recent comparative studies have shown good results for most of the psychometric properties of the iHOT12 [4, 9, 12, 15, 36]. Our study also showed a high level of reliability and validity for the German version of the iHOT12.

### Study design and population

Our demographic data are comparable to other studies on young active patients generally undergoing hip arthroscopy with an average age around 35 years [6–8, 15, 32, 33]. In order to get a more heterogeneous patient sample, we did not preselect patients according to their diagnosis or intended treatment. Aiming for validation data for various hip diseases, we included all patients with a hip disorder and an activity level greater than 4 on the modified Tegner activity scale (mTAS). Compared to the original publication of the iHOT by Mohtadi [9] we chose more liberal inclusion criteria sparing a limitation of age. We nonetheless excluded patients with a disorder of the back or the contralateral lower extremity or a mental disorder to avoid confounding. The number of patients included in our study is according to previous recommendations [25].

The translation process was conducted according to the guidelines of the American Academy of Orthopedic Surgeons (AAOS) Outcomes Committee [17]. We chose the period of time between test and retest to be a minimum of two weeks as recommended in the COSMIN checklist [24]. The validation was carried out following the complete COSMIN checklist [13, 24]. Along with the prospective multicenter design, the study meets high methodological standards with a level of evidence Ib.

### Reliability

The good correlation coefficient for Cronbach’s alpha outlines the quality of the German iHOT12 and confirm the results of prior validation studies on the iHOT [9, 12, 15, 36]. Accordingly, an ICC of 0.94 confirmed excellent test-retest reliability. Low values for measurement error and smallest detectable change (SDC) indicate that small clinical changes can be detected not only at group level but also at the individual level [25].

### Validity

For the evaluation of the construct validity, the HOS and EQ-5D seemed most appropriate because they are applicable in this patient population and validated in the German language [20, 22, 23]. The HOS was also developed for patients undergoing hip arthroscopy [20]. Therefore, it seemed a viable instrument to evaluate construct validity. We determined a sufficient level of activity by including only patients with an activity level greater than four on the mTAS as described by Mohtadi et al. [9]. Because the mTAS is solely derived from the level of sports activity, it is rather robust to smaller changes of the medical condition. Therefore, we expected a rather poor correlation between the functional hip scores and the mTAS.

Griffin et al. [12] proved similar characteristics of the short version (iHOT12) compared to the extended version (iHOT33). The excellent correlation between the iHOT12 and the iHOT33 in our study supports their assumption that for clinical use, the 12 questions of the short version are sufficient.

### Responsiveness and interpretability

In our population, the minimal important change (MIC) according to the method of Norman et al. [28] was 13.79. There was a larger effect of the difference of iHOT score and the HOS score between the groups shown in Table 1. This suggests that even with only 12 questions the iHOT12 seems to have a high discriminatory power. This indicates a reasonable ability to transform a qualitative effect into a quantitative. Using the method by Norman is controversial in literature [4, 7, 21, 37, 38]. Norman et al. [28] suggested an estimation of the MIC by division of the standard deviation by two. This method was derived from the effect size when undergoing clinical intervention. Based on Terwee et al. [25] a positive rating for responsiveness can be assumed when SDC is greater than MIC. With an anchor based method the patient’s report a current state of health targeting for their personal expectations [4, 37, 38]. Therefore, calculation of MIC can be problematic in absence of a therapeutic gold standard. The COSMIN checklist does not contain any recommendations on the estimation of the MIC. Still, there is no consensus about how to assess the MIC. Although the 95 % CI of the “unchanged” and “somewhat better” group are wide, a remarkable difference exists only within a small range of 0.18-0.55. The cut-off point that separates the “unchanged” group from the “somewhat better” group has to be outside of the 95 % CI of the “unchanged” group. It should also represent the smallest acceptable effect size for the “somewhat better” group. Accordingly, an estimated ES of 0.55 seems reasonable to approximate the true ES of the iHOT12. Consequently, the minimal important change that reflects a clinically relevant improvement is less than 14 points on the iHOT12. Defining the MIC of the iHOT12 is a main aspect of this study.

Our findings according to the global rating of change also confirm a strong correlation between patient perception and score result of the iHOT12.

### Limitations

Despite good results concerning validity, reliability, and responsiveness of the iHOT12, there are some limitations to this study. Due to a lack of a therapeutic gold standard, the minimal important change could only be estimated. To date, the only study providing longitudinal results on the iHOT is Mas Martinez et al. [8]. They report on short term outcomes after hip arthroscopy in FAI with a minimum follow up of 12 months. Unfortunately, data concerning minimal clinically important change are not specified in this study. Further prospective studies on longitudinal measurement properties of the iHOT12 are needed.

## Conclusion

The German version of the iHOT12 provides good validity, reliability, and responsiveness for the functional evaluation of physically active patients with a hip disorder. This is the first study to define the minimal important change (MIC) which less than 14 points on the iHOT12 scale. The COSMIN checklist is a feasible guideline to assess psychometric properties of patient reported outcome measurements.

### Ethics, consent and permissions

All patients had given their written informed consent to participate in this study. The Regensburg University Ethics Committee approved the study in November 2013 (Institutional Review Board Number 13-101-0259).
